# Glans Ischemia after Circumcision in a 16-Year-Old Boy: Full Recovery after Angiography with Local Spasmolysis, Systemic Vasodilatation, and Anticoagulation

**DOI:** 10.1055/s-0038-1667330

**Published:** 2018-09-28

**Authors:** Richard Gnatzy, Jochen Fuchs, Manuela Siekmeyer, Anne Bettina Beeskow, Jan-Hendrik Gosemann, Martin Lacher

**Affiliations:** 1 Department of Pediatric Surgery, University of Leipzig, Leipzig, Germany; 2 Department of Diagnostic and Interventional Radiology, 310 Klinik GmbH, Nuremberg, Germany; 3 Hospital for Children and Adolescents, University of Leipzig, Leipzig, Germany; 4 Department of Radiology, University of Leipzig, Leipzig, Germany

**Keywords:** Glans Ischemia, children, digital subtraction angiography

## Abstract

Circumcision is one of the most frequent procedures performed by pediatric surgeons. A dorsal penile nerve block (DPNB) is commonly used for perioperative analgesia. We report the case of a 16-year-old boy with ischemia of the glans who presented on the first postoperative day after circumcision under DPNB (0.25% bupivacaine) at an outside hospital. The patient underwent immediate angiography under sedation. An intra-arterial spasmolysis was performed with alprostadil and nitroglycerine and a sufficient perfusion of the glans penis was confirmed. Subsequently, systemic sildenafil, arginine, and heparin were given. Following this 3-day medical treatment, ischemia resolved completely. Our case emphasizes the role of invasive angiography in the diagnostic workup and the therapeutic possibilities of local spasmolysis, systemic vasodilatation, and anticoagulation.

## Introduction

Circumcision is one of the most frequent procedures performed by pediatric surgeons. However, after surgery some patients represent to the emergency department due to bleeding, pain, swelling, redness, local infection, and decreased urinary output. Most of this morbidity is minor and can be easily managed. 1 In contrast, ischemia or even necrosis of the glans is a rare complication; in the past 15 years, nine pediatric cases have been reported in the literature. 2 3 4 5 6 7 8 We report the case of a boy with ischemia of the glans penis after circumcision at an outside hospital.

## Case Report

**Fig. 1:**
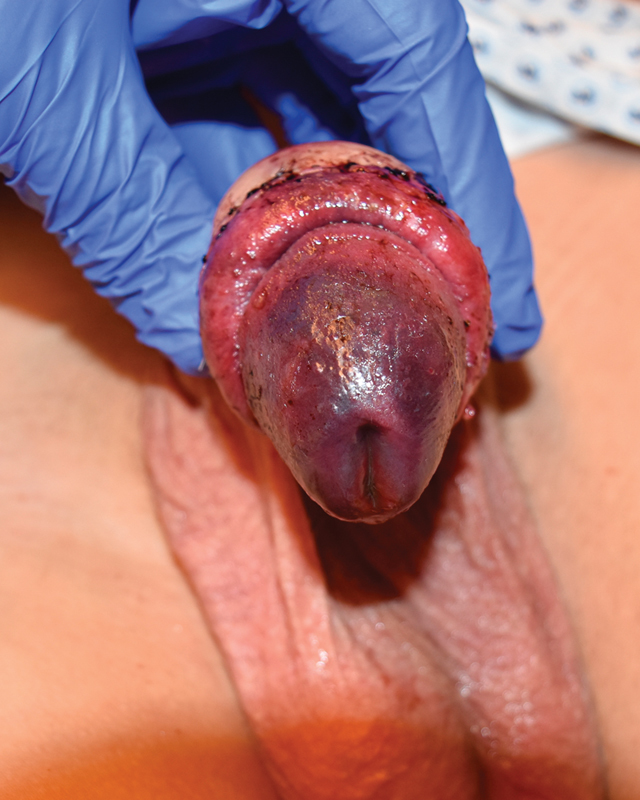
Glans with severe ischemia on the first postoperative day (admission).

**Fig. 2:**
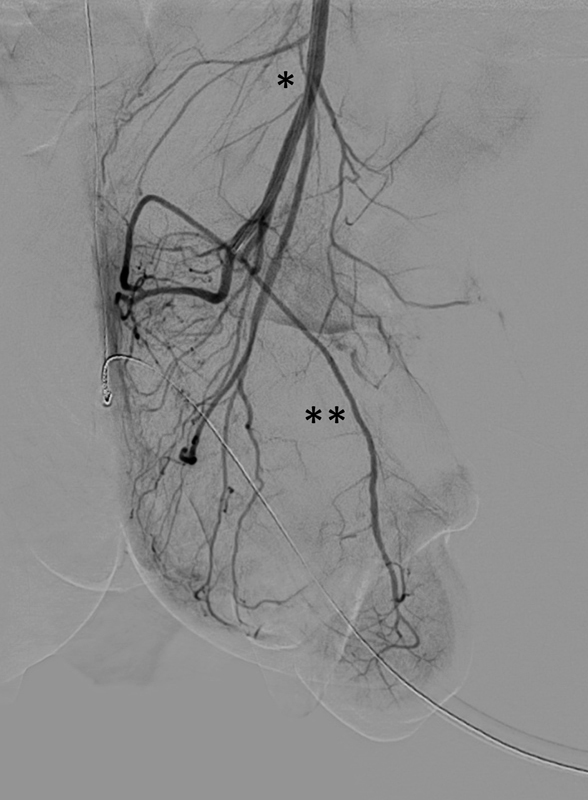
Digital subtraction angiography (DSA) of the left internal pudendal artery (*) via microcatheter (Progreat 2.7F, Terumo) confirmed a sufficient arterial perfusion of the glans with good contrast filling of the dorsal artery of the penis (**).

**Fig. 3:**
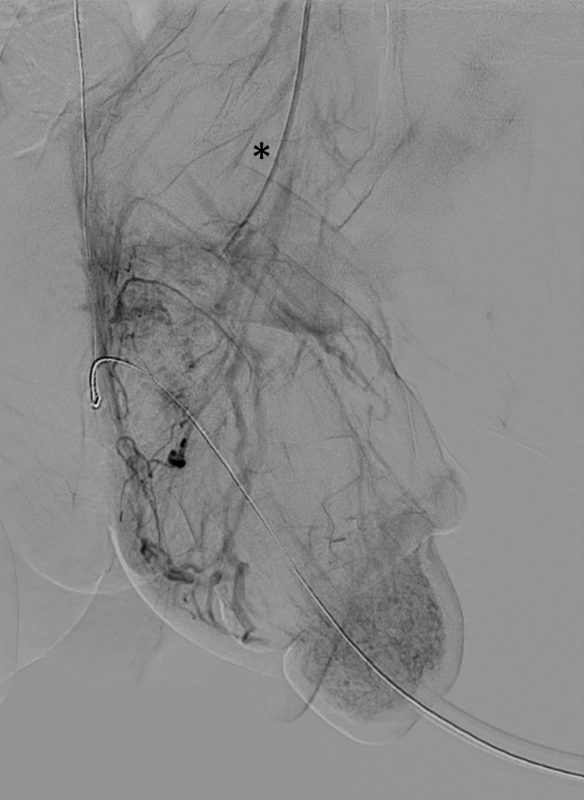
Venous phase of the digital subtraction angiography (DSA) of the left internal pudendal artery (*) showed a sufficient venous drainage of the glans.

A 16-year-old boy with phimosis underwent elective circumcision at an outside hospital. Anesthesia was performed via dorsal penile nerve block (DPNB) (15 mL of 0.25% bupivacaine). On the first postoperative day, he was referred to our hospital due to pain, black discoloration, and swelling of the glans. Voiding was possible. On clinical examination, the distal glans showed severe ischemia ( Fig. 1 ). All laboratory results including blood count, lactate, D-dimer, and clotting profile were within normal limits. Color Doppler ultrasound of the penis showed good cavernosal artery flow to the glans. After transferral to our pediatric intensive care unit, a caudal block was performed to reduce sympathetic tone and improve arterial blood flow. Five hours after admission, the patient underwent digital subtraction angiography (DSA) under sedation. After overwiew of the pelvic arteries and the left internal iliac artery, the internal pudendal artery was explored selectively via microcatheter (Progreat 2.7F, Terumo) but no vasospasm or thrombus was detected. A sufficient arterial perfusion as well as normal venous drainage of the glans was confirmed ( Fig. 2 and 3 ). To use all therapeutic options, intra-arterial spasmolysis with a bolus of 5 µg alprostadil and 150 µg nitroglycerine was sequentially given. Eight hours after admission, systemic therapy with sildenafil (1 mg/kg orally once a day), L-arginine-hydrochlorid (0.1 mg/kg/hour), and unfractionated heparin (15 units/kg/hour, up to 20 units/kg/hour depending on partial thromboplastin time) were initiated and given for 3 days.

**Fig. 4:**
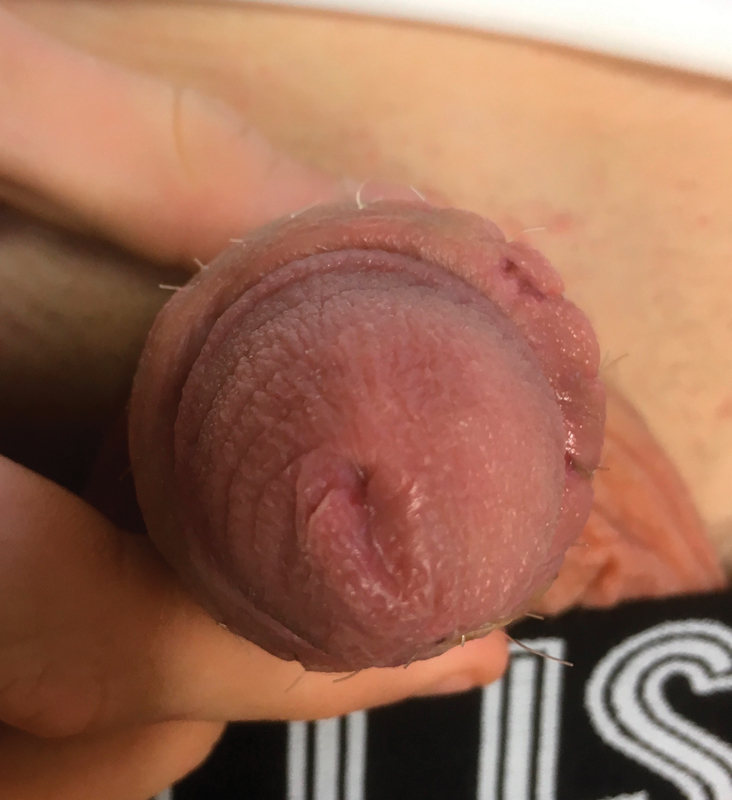
After treatment, glans ischemia resolved completely (seventh postoperative day after angiography).

After 3 days of systemic vasodilatative therapy, the darkish color of the glans changed to a brownish appearance. A surgical intervention was not necessary and ischemia resolved completely. The boy was discharged on the seventh postoperative day without adverse events ( Fig. 4 ).

## Discussion

Glans ischemia is an extremely rare complication after circumcision. In most cases, the etiology remains unclear. Apart from hematoma, tight suture lines, or excessive use of monopolar cautery, DPNB has been suggested to be the most frequent cause of this complication. 4 9 In a large cohort from Singapore analyzing 3,909 DPNB, the total complication rate was reported to be as low as 0.23%. 10 Other authors also report on ischemia of the glans penis following DPNB for circumcision. 6 7 11 As an alternative for DPNB, topical anesthetics have been evaluated but did not provide sufficient perioperative analgesia. 12 Kaplanian et al suggested to limit the volume of local anesthetic solution to an amount of 0.2 mL/kg (up to maximum of 10 mL). 7 In our patient, the primary surgeons exceeded this dose and applied 15 mL of plain bupivacaine on each side. Therefore, this could have led to a transient vasospasm.

In case of glans ischemia following DPNB, the main goal of treatment is the reestablishment of sufficient blood flow to the penis. Several therapeutic approaches have been described including pentoxifylline, hyperbaric therapy, iloprost, enoxaparin, anticoagulation, local testosterone, and peridural anesthesia. 2 3 4 5 6 7 8

In our case, we started treatment by performing a caudal epidural block to improve arterial supply and to reduce sympathetic tone and pain. This is in line with a report of a group from Australia, who successfully applied this technique in a 9-year-old boy. 7

Thereafter, we focused on treating the suspected vascular origin of glans ischemia. In adult urologic departments, DSA is frequently used in the diagnostic workup for arteriogenic impotence and percutaneous endovascular revascularization to treat vasculogenic erectile dysfunction (penile artery stenosis) 13 14 15 or in the treatment of high-flow priapism. 16 To the best of our knowledge, we applied invasive angiography for the first time in a patient with glans ischemia. We observed a sufficient arterial perfusion as well as a normal venous drainage of the glans. A vasospasm or arterial obstruction, as well as a venous outflow problem, was ruled out during this procedure. Additionally, potent vasodilators nitroglycerine (nitric oxide as cyclic guanosine monophosphate activator) and alprostadil (prostaglandin E1 analog) were applied intra-arterially to test if this could further improve the perfusion, which was not the case.

The nonselective phosphodiesterase inhibitor pentoxifylline (PTX) is a hemorheological drug, which improves peripheral blood flow by reducing whole blood viscosity. It works by relaxing smooth muscle of the corpus cavernosum. Two case reports on a 3- and 10-year-old boy combined the therapy of caudal block with PTX and described good outcomes. 4 6 However, indication and dosage of PTX in children remain controversial. 3 4 6 8 11 Hence, in our patient we used the selective phosphodiesterase inhibitor (PDE 5) sildenafil as a vasodilative agent with success and did not appreciate side effects.

Efe et al suggested a monotherapy with low molecular weight heparin (enoxaparin) as the treatment of choice for ischemia of the glans in a 7-year-old boy. After 5 days of anticoagulation, the black discoloration of the glans disappeared. 5 In our patient, we used the protocol of unfractionated heparin (15–20 units/kg/hour) according to the protocol of Sara and Lowry ([bupivacaine 0.5%] with low-dose heparin infusion [25 units/kg/hour] for 4 days). 9

Other therapeutic options include hyperbaric therapy, which has been evaluated in adults only. 17 Therefore, we did not consider this therapeutic option.

## Conclusion

Ischemia of the glans is a rare complication after circumcision after DPNB. Although the cause of the transient ischemia could not be proven, we speculate that DPNB might have caused severe vasospasm. In the current patient, the multimodal treatment resulted in complete recovery of the glans without adverse events. Our case emphasizes the role of DSA in the diagnostic workup and the therapeutic possibilities of local spasmolysis, systemic vasodilatation, and anticoagulation.

We acknowledge support from the German Research Foundation (DFG) and Leipzig University within the program of Open Access Publishing.
